# HIV-positive migrants’ experience of living in Sweden

**DOI:** 10.1080/16549716.2020.1715324

**Published:** 2020-01-30

**Authors:** Manijeh Mehdiyar, Rune Andersson, Katarina Hjelm

**Affiliations:** a Department of Infectious Diseases, Institute of Biomedicine, The Sahlgrenska Academy, University of Gothenburg, Gothenburg, Sweden; b Departments of Clinical Microbiology, Sahlgrenska University Hospital, Gothenburg, Sweden; c Department of Public Health and Caring Science, Uppsala University, Uppsala, Sweden

**Keywords:** HIV-positive, migrants, life situation, Sweden, content analysis

## Abstract

**Background**: There is a limited knowledge of the impact of being human immunodeficiency virus (HIV)-positive on migrants living in Sweden. It is therefore important to gain a general awareness of this issue in order to maintain the wellbeing of this vulnerable group of patients and to develop an adequate social support network.

**Objective**: The aim of this study was to explore HIV-positive migrants’ experiences of their life situations, living in Sweden.

**Method**: A qualitative, exploratory study was performed using semi-structured interviews with 14 HIV-positive migrants, aged 29–55 years, and analyzed with qualitative content analysis. The participants were recruited from three clinics for infectious diseases in western Sweden.

**Results**: The results are presented in the following three categories: ´Vulnerability in social relationships’, ‘Fear of disclosure”, and *‘*Resilience’. The results illustrated the participants’ experiences of vulnerability in their social relationships, fear of disclosing HIV status, feeling lonely and stigmatized, and lacking social network and support. Furthermore, the results illustrated participants’ challenges in finding a partner, due to their fear of being recognized because of their HIV-infection. However, the result indicated participants’ struggling for a normal life with integrity, and that their need to look positively at life.

**Conclusions**: In the actual study loneliness, fear of disclosure, perceived stigma, and the lack of a social network had significant impact on the life situations of the HIV positive migrants. Fear of disclosure and the challenge of finding a partner and friends were the main obstacles. It is crucial to increase access for these patients to supporting networks that will promote their empowerment and trust.

## Background

Due to the improved treatments, the human immunodeficiency virus (HIV) infection has now become a chronic disease [1]. Recent studies have also shown that HIV-positives’ life expectancy is approaching that of the general population [2]. However, the life expectancy of the most vulnerable group of people living with HIV, such as non–white people, and those with a history of drug use has not yet improved to the same extent [2].

**Table 1.** The sociodemographic characteristic of the study participants

	Level of education			Age (years)			Occupation		Number of years living in Sweden			Region of origin			
Participants	Primary school	High school	University	29–39	40–49	50–59	Employed	Unemployed	1–7	7–14	15–20	Africa	South East Asia	South America	Eastern Europe
Women N = 7	2	5	0	2	3	2	3	4	2	4	3	5	1	1	0
Men N = 7	1	5	1	2	4	1	4	3	1	2	2	5	0	1	1

**Table 2.** Example of analytical process

Meaning unit	Code	Sub-category	Category
It would have been nice if I had someone to talk to at least during the first months after my diagnosis.	Absence of contact and support	Lack of social network and support	Vulnerability in social relationships

Migrants living in Europe are a diverse group with varying degrees of vulnerability [3].

The term migrant includes a wide variety of people with different backgrounds and life situation. It includes both forced and voluntary migrants [4]. Forced migrants (refugees and asylum seekers) are people who are forced to move from their home as a consequence of war, persecution, poverty, lack of human rights, environmental disasters etc. Voluntary migrants (immigrants) often leave their home in search for better work and living conditions [4].

A systematic review showed poor self-perceived health among migrants and ethnic minority groups in Europe compared to the majority population [5]. Social processes shaped by socio-economic status have been proven to be the main causes of vulnerabilities among migrants globally [6]. On the other hand, various studies from high-income countries, among others USA, Canada, UK, and Australia show a ‘Healthy immigrant effect’ which refers to the evidence that migrants who have recently arrived in the country have, on average better health compared to native-born people, but their health status is diminished after a time following immigration [7–10]. However, refugees who spend time in refugee camps generally experience a health disadvantage upon arrival in the community [11].

Generally, there is a need to improve the general awareness of the social determinants of health on migrants’ life context [12]. The World Health Organization defines the social determinants of health as the circumstances in which individuals are born, grow, live, work and age, which are mostly responsible for health inequities [13]. This is crucial since, living with HIV is closely connected to public stigma. Public stigma is the response of society to stigmatized individuals based on the negative and discriminative attitudes [14]. The latest report from United Nation Aids [15] showed that fears of HIV infection and prejudices against people living with HIV persist despite decades of public information campaigns and other awareness-raising efforts.

The Swedish Communicable Diseases Act and the Communicable Diseases Ordinance [16] classifies HIV as dangerous to public health and to society and subject to mandatory contact tracing. As a result, people living with HIV have the obligation under the criminal law to disclose HIV status to their sexual partners, which increases the vulnerability of these people in their social life. However, there are a number of governmental and non-governmental organizations (NGO) in Sweden that provide different kinds of social support to people living with HIV.

Since the start of the HIV epidemic, HIV-infection has been closely linked to stigma and discrimination [17]. On the other hand, stigmatizing attitudes are strongly correlated with a misunderstanding of the mechanism of HIV transmission and an overestimation of the risk associated with casual contact, and with the negative attitudes towards vulnerable groups who are disproportionately affected by the epidemic [18]. For instance, perceived stigma and discrimination have caused avoidance or delay in seeking health care among HIV-positive African women living in Belgium [19]. Perceived stigma refers to the individuals’ awareness of public stigma [14]. Another study found that women’s non-disclosure functioned as coping, and this made them feel resilient [20]. A doctoral thesis of HIV-positive African parents living in Stockholm, showed that these African parents had a limited network with few people who they could talk to about their HIV status, and that they mainly sought to avoid disclosure of their HIV status to their children, relatives and friends [21]. Another study from Canada showed that a combination of structural barriers in terms of access to employment for immigrants together with the HIV-related stigma resulted in the marginalization of the HIV-positive Latino community [22].

Our previous study of ‘HIV-positive migrants’ encounters with the Swedish health care system’, demonstrated that free access to antiviral therapy was necessary but was not enough for them to feel satisfied with the Swedish health care system [23]. In another study on Swedish HIV caregivers, access to information about the Swedish health care system and its functions, as well as the right to have access to information about the Swedish welfare system and its complexities, were found to be essential for migrant patients to obtain access to these rights [24]. Furthermore, there is a lack of studies relating to the life experiences of migrants living with HIV in Sweden, and this needs to be addressed.

The aim of this study was to explore HIV-positive migrants’ experiences of their life situations, living in western Sweden.

## Method

### Study design

A qualitative exploratory study design with semi-structured interviews for data collection [25] was chosen in order to gain an understanding of the life experiences of HIV-positive migrants living in Sweden.

### Participants

The participants were recruited through purposive sampling. The inclusion criteria were HIV-positive migrants being registered at the clinics for infectious diseases in a region of Sweden. Permission to contact and recruit HIV-positive participants for the study was obtained from the heads of the clinical departments. The study participants were contacted and recruited between 2011 and 2014 at three outpatient clinics of departments for infectious diseases, in hospitals in Sweden. The patients who visited the outpatient clinics were invited to participate in the study by the nurses, during the period of study recruitment. During their visit to the clinic, all HIV-positive migrants were given information about the study by the nurses in English or Swedish, or by interpreter. Patients who agreed to participate in the study were able to contact the researcher personally by telephone or through the nurses at the outpatient clinics. The majority of contacts were made through the nurses.

The participants included seven women and seven men, aged between 29 and 55 years with a median age of 37, who had been living in Sweden for between 2 and 20 years. They had different backgrounds, education, and socioeconomic status (Table 1). It was spontaneously disclosed in the discussions, when the participants talked about their partners that all participants with the exception of one man identified themselves as being heterosexual.

### Data collection

The interview guide was based on the research questions and was reviewed by all authors. A pilot interview was conducted before the interviews, and the interview guide was revised. The interviews were led by the first author who is a social worker, with experience in implementing qualitative interviews. This author has worked with HIV prevention at an administrative level in the community. The interviews covered the following three main themes:

How do you perceive your life situation in Sweden?
How would you perceive your relationships with your family and friends?
What difficulties have you experienced in the new community?

The questions provided the framework for the actual content of the interviews and were followed by more extensive, in-depth discussions including more probing, follow-up questions [25]. The sample size of the study was determined on the basis of the information required to answer the research questions with sufficient certainty. Data collection continued until analysis of data indicated that no further information had been added [25,26]. The participants chose a place that was convenient for them for the interviews to take place. Eleven participants chose to be interviewed in the outpatient clinic, one in his home, and two at a coffee shop. Eleven interviews were conducted in Swedish, two in English, and one with the assistance of a Somali-Swedish interpreter. The interviews lasted between 40 and 60 min. All interviews were tape-recorded and transcribed verbatim by the first author.

### Data analysis

The analysis of data proceeded simultaneously with data collection according to content analysis, and continued until it indicated that no further information had been added [25]. The interviews were listened through and transcribed verbatim. The interview text was read several times in its entirety and was analyzed for context in relation to the aim of the study to provide a comprehensive picture of the study context. The context is important in all analytical methods, but this is emphasized in the content analysis as being central [25]. The analysis of the data involved identifying the content by means of differences and similarities. To enable this, textual units were identified; content was named with codes, and was then sorted into sub-categories according to their relationships (Table 2). Similar sub-categories were sorted into categories and named as close to the text as possible to reflect their content.

Measures were taken to increase validity of the data [25], and these were as follows: the interviews were read and their contexts were analyzed by the first author, and the contents of the categories were checked by the co-authors. One of these was a researcher in nursing science with focus on migration and health and the other a researcher in infectious diseases experienced in the care of people with HIV. The researchers regularly checked and discussed the content of the categories and a comparison of these showed a high level of agreement. Quotations from the interviews are presented in the text to provide support for the different categories of the data [25].

## Results

**Figure 1.**
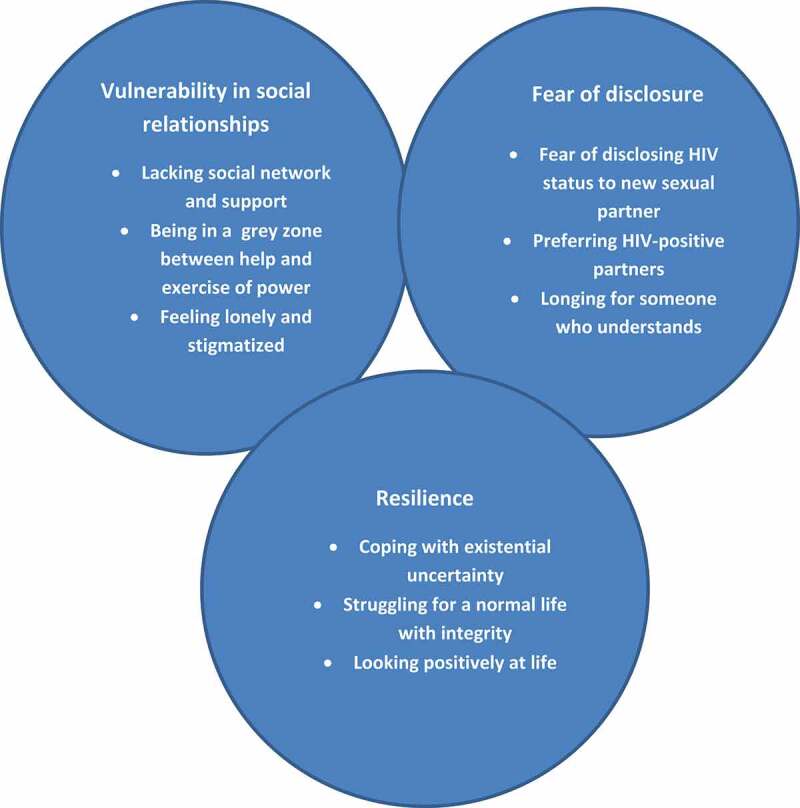
The three main categories with their supporting sub-categories

The analysis resulted in three main categories with supporting sub-categories as illustrated in Figure 1. The results are presented under the headings of the categories with the supporting sub-categories as sub-headings.

### Vulnerability in social relationships

The participants’ experiences of isolation, stigma and the lack of a social network and support have made them vulnerable in their social lives. The majority of the study participants were originally from Africa. However, socioeconomic status, sexual orientation, gender, and family relationships determined the degree of vulnerability as well as the need for access to a social support network. For instance, a homosexual HIV-positive man who had moved to Sweden due to marriage to a Swedish partner had less experience of vulnerability in social relationships compared to an African woman living alone with her child in Sweden.

#### Lacking social network and support

The participants experienced their need for access to a social network and support. However there were differences between participants’ experiences of this need depending on age, gender, sexual orientation, and socio-economic status of the participant.

*‘So I was quite sad then for a long period of time. I did not know how long I could live and I thought about the family and if I could have children and all that. And I could not talk to dad about such stuff as usually boys and father usually do not have such contact. And I was not good enough at the Swedish language to be able to get in touch with a social worker and such stuff. So it would have been nice if I had someone to talk to at least during the first months after my diagnosis because I was quite young … later, I was asked if I could go to Stockholm to meet others who are HIV-positive, but I did not feel safe then, I was only 17 years old then.’* [P6]

*‘I have thought quite a lot if there could be somewhere you could meet someone in the same situation*.’ [P2]

#### Being in a gray zone between help and exercise of power

The participants described their experiences of vulnerability related to the different social actors’ power relationships. They felt that some actors lacked both knowledge about HIV as well as an understanding of the participants’ social context which, in turn resulted in suffering for the participants.

*‘I had two children then when I was told that I had HIV-infection in Sweden. And how I could deal with that … And the social office demanded that I must tell my children about my HIV-infection. And I said but why? Why should I tell my children that I have HIV? Oh dear, oh. They wanted it and I said no, I definitely do not do that, I said. You guys have nothing to do with that and they said no and it was such a fight. Why, why, why do my children need to know? For what? Oh my god, it was such a psychic pressure. I felt so bad that I wanted to die. Oh my God.’* [P7]

*‘When they tested me and they found that I am HIV- positive, they just told me to go and tell my partner that I was HIV-positive. But I didn’t want to tell her. It was so difficult to tell her. Because I knew when I would tell her, she just started discriminating against me.’* [P1]

#### Feeling lonely and stigmatized

The participants experienced great loneliness due to perceived stigma as a result of fear of disclosure of HIV. The experience of loneliness and perceived stigma is described as being related to the fear of being recognized as HIV-positive by people from their home country as well as by Swedish people.

*‘I don’t dare to let people (from my home country) into my life. They talk too much so I don’t dare to let them in, because then I’ll be afraid to do this and that. For example, when my boy was little I could not breastfeed him and so everyone comes and looks and wants to congratulate my baby and so I was afraid (to be recognize as HIV-positive) when I had to give him baby food instead of breastfeeding. Yes, in Africa it is normal for women to breastfeed, but in Sweden there are many who do not breastfeed.’* [P3]

*‘Because you are so afraid to tell about yourself, you reject many people and then the only thing left is the job you will be looking forward to … And the life becomes completely strange and you get frustrated.**’*** [P6]

### Fear of disclosure

This category revealed the participants’ experiences of fear of disclosure of HIV status and the consequences of this. The respondents report that they were not living openly with HIV, and they often expressed difficulties in finding a partner, or close friends because of their fear of disclosure, which, in turn had caused a strong desire for a partner and close friends.

#### Fear of disclosing HIV status to new sexual partner

The majority of the participants were single and they expressed difficulty in finding a partner. Almost all of the participants expressed uncertainty and a fear of telling the new partner about their HIV-infection.

*‘I had a friend and I tried to be open and told her (about my HIV-infection) but she disappeared and then I was afraid, I don’t dare to tell anyone about my HIV-infection anymore. I have tried to date sometimes, but I have not managed to tell about my HIV-infection, because when I say about it people just run away.’* [P3]

*‘If you want to get a girlfriend or children and stuff like that, you have the obstacle of being HIV-positive. You cannot do like everyone else. You have to tell your partner before sexual relationship … because you don’t know how they are going to take it. And it makes you live a little lonely.’* [P6]

#### Preferring HIV – positive partners

The participants expressed a desire for an HIV-positive partner. Finding a partner was something they desired yet found difficult because of their fear of discrimination and rejection from a non-HIV-positive partner.

*‘It is much better maybe to meet a girl who also has HIV to live together with. Sometimes I wonder how I could find a girl who also has HIV. She may have the same problem as me and is alone, so we can live together. But with some-one who is completely healthy I do not know if I can get. I think it was much better maybe to meet a girl who also has HIV-infection and to live together. Because she also has HIV-infection, there is nothing to be ashamed of or afraid of … But how can I fix it? I have had partners but because they were not HIV-positive, they were not satisfied to live with someone who is HIV-positive.’* [P2]

*‘I wish I could live in a marriage with someone as well, with someone in the same situation (HIV-positive). I don’t know how to get to know someone in my situation. I have tried, I have been searching on the internet but nothing has happened*.’ [P3]

#### Longing for someone who understands

Fear of disclosure has caused loneliness and a lack of close friends for the participants. They expressed a desire and longing for close friends or someone in their private life who they could share their experiences with.

*‘What I miss most is someone to talk to. Someone very close, not health care staff. I can talk to you, but you do not really understand. I can talk to my social worker but she does not really understand how it is. It should be someone in the same situation’*. [P7]

*‘Before, we could meet a psychologist (in the NGO). We were not many, only four people and we met one time a month, but it was good, it was empowering to meet others and to share experiences with others … But now we have no-one to meet.’* [P4]

### Resilience

The category, ‘Resilience’ describes the participants’ experiences of strength in spite of their vulnerability in living with HIV.

#### Coping with existential uncertainty

The participants described their everyday fear of living with a chronic disease that not only demands lifelong treatment and uncertainty but also involves coping with existential uncertainty, the new identity and a lifelong chronic disease.

*‘We have been prejudiced against HIV when we were not HIV-positive ourselves. And when we get the HIV-diagnosis, the most difficult thing is to say that now it’s about me. Before, it was about others, friends or some-one else, but now it’s about me and how am I going to live with this? When I found out that I am HIV-positive, I have to know what is going to happen with me, why we are going to live longer than before, how many medicines we have now and how many in 10 years’ time. I just try and understand that disease a bit better.’* [P11]

*‘I’m not depressed or so, but I’m not the same person as before (HIV-diagnosis). I do not live with the same security as everyone else.’* [P5]

#### Struggling for a normal life with integrity

The participants expressed a desire for, and the struggle with living a normal life like every-one else, with independence, respect and integrity without being treated differently by others because of their HIV-infection.

*‘I always tell myself to get up and go. Do not sit there and think it’s a pity for me, I tell myself. No, get up and go, because there is always someone who is worse off than you, always.’* [P7]

*‘It makes a big difference to me that people accept me as a human, not as just HIV-positive.’* [P6]

#### Looking positively at life

To be grateful, to think positive, and look at the bright side of life, and to compare themselves to those who are in a worse situation, these things gave the participants strength. This was important as a basis for coping and as a source of resilience for the participants.

*‘And I always think so that there’s always somebody who’s worse off. And there are many other diseases like ALS, Parkinson that are worse. For example, I can walk, I can work, I can feed myself. I think about those who have diseases that there is no medicine for. To me, I’m just grateful for each day, I’m grateful. I appreciate life differently. Things I thought were important before are no longer important to me. Nothing to complain about, nothing. Thanks.’* [P7]

*‘But it’s always a new phase, one has to get used to it and think that in a good way, thanks to the medicine. Think, it always depends on which way you look at your life, in a brighter or darker way.’* [P11]

### Discussion

The results of this study are unique as we explored the HIV-positive migrants’ life experiences, illustrating fear of disclosure, loneliness, perceived stigma and the lack of a social network. It showed the challenges that they face in finding a partner and friends due to their fear of being recognized because of their HIV-infection. Furthermore, the study illustrated participants’ struggles to live a normal life with integrity, and to look positively at life. Socioeconomic status, education, and background determine the varying degrees of vulnerability in social relationships. For instance, the study of stigma experienced by HIV-positive women in Ontario, Canada showed that women with an African background had a greater vulnerability, because of structural racism and discrimination [27]. Thus, the intersections of the different forms of stigma need to be studied further in the context of HIV since these intersections synergize the level of stigma, which exposes those affected to a greater degree of vulnerability [28–30]. The report from The Committee on Migration, Refugees and Displaced Persons of the Council of Europe [31] showed that, for migrants living with HIV in Europe the difficulties in accessing housing and employment provided further obstacles to their coping than the actual HIV diagnosis.

The fear of disclosure experienced by the participants was another category within the study, and this resulted in difficulties in finding a sexual partner, friends or building a social network. A systematic review on HIV disclosure among African immigrants in Europe [32] found that stigma and other social factors such as fear of rejection, abandonment and isolation are associated with avoidance of HIV-disclosure. Further, it showed the advantages of HIV-disclosure in terms of mental and physical health with a decreased anxiety when a burden of secrecy is removed [32].

Longing for an HIV-positive partner was the sub-category that referred to the challenges faced by the participants related to the difficulties of having a partner due to HIV-disclosure. A study conducted in Sweden showed a positive correlation between access to counseling, social networking, and HIV-disclosure [33]. Social support through social networking has been shown to play a major role not only in empowering people living with HIV, but also in HIV-prevention. Several studies illustrate that migrants with access to social networking and support within their communities are less at risk of HIV- infection [34–36]. Social support includes both material and non-material support such as access to information, facilitation, and also emotional support during times of hardship [37,38].

The study also describes the participants’ experiences of resilience in coping with existential uncertainty, their struggle to live a normal life with integrity and to look positively at life. Resilience is described as the ability to overcome difficulties and recover from adversity [39]. Even though evidence points to the fact that HIV infection has an unique impact on physical, psychological, and social well-being, various studies have reported that people living with HIV are classified as being both vulnerable and resilient [40]. This shows that physical, mental and social vulnerability caused by HIV infection have resulted in a resilience among many individuals which, in turn helps them to cope with the disease or their life situations and to lead a dignified life. For instance, in the community of HIV-positive Sub-Saharan African women living in Switzerland, even though HIV-disclosure is highly stigmatizing, these women choose disclosure and non-disclosure on the basis of their individual rights and obligations. This illustrates that some women choose disclosure in order to defy racism and discrimination [41]. The same study also showed that these women were not only victims but also agents in their own lives [41].

Sweden has succeeded in enhancing HIV-care, and has become one of the few countries to achieve the United Nation Aids/World Health Organization ‘90–90–90 target’ [42]. This means that, by the end of 2015, 90% of all HIV cases in Sweden were diagnosed. Of these, 99.8% were undergoing HIV-care, and in 95% of treated cases, HIV viral load was undetectable [43]. On the other hand, the participants’ fear of disclosure along with the provisions of The Swedish Communicable Diseases Act, which obliges them to inform prospective partners under criminal law, increases the vulnerability of these people to the stigma and discrimination [44]. In addition, the participants’ lack of social support described in this study shows that social support services to migrants living with HIV need to be improved in order to increase accessibility, and acceptability of social support among migrants living with HIV. Adapted social support and social networks are therefore crucial in order to reduce structural barriers, such as language and cultural barriers, and promote resilience and empowerment among migrants living with HIV in Sweden.

Although further studies are needed to expand our understanding of the different intervention approaches, the results of this study should be considered by the different professions and civil authorities involved. In this study, we did not discuss gender differences as this represents the first explorative step towards extending our understanding of HIV-positive migrants’ experiences of living with HIV in Sweden. This is a limitation which needs to be studied further. The first author had experiences of working with HIV-prevention at the structural level, and this has given her a certain preunderstanding of the studied area that might serve to influence the analysis of data. However, this was minimized by analyst triangulation [26] with the co-authors involvement in the process of data analysis.

Since qualitative research is context bound, the result of this study may be transferable for a similar group in a similar context, as it has been carefully designed, conducted, and analyzed [25] in this study. The participants in this study were not living openly with HIV, and the experiences might differ from those of migrants who are living openly with HIV or migrants who have access to adapted social support services or networks. However, qualitative studies on migrants living with HIV are limited and this study may serve as a base for future studies.

## Conclusion

The findings of this study demonstrate that perceived stigma, fear of disclosure and the consequences of these, have limited the participants’ chances of finding a life partner, friends and access to a social network. Further, the lack of an adapted social support and network has left migrants living with HIV vulnerable in their social lives. They experienced vulnerability related to their HIV-infection with different social actors, due to the actors’ power relationships. There is a need to reduce the perceived stigma and discrimination that migrants living with HIV experience in different ways in order to enhance their well-being. In this respect, it is crucial to increase access for these patients to support networks that will, in turn promote their empowerment and trust.

## Paper context

It is known that migrants living with HIV in Europe are vulnerable with varying degrees of vulnerability. In the actual study, perceived stigma, fear of disclosure, loneliness, the challenge of finding a partner, friends, and the lack of a social network had significant impact on the life situations of migrants living with HIV in Sweden. A prioritization of the social vulnerability of migrants living with HIV is needed by policy makers and social actors.
